# Dietary Phospholipids Prepared From Scallop Internal Organs Attenuate the Serum and Liver Cholesterol Contents by Enhancing the Expression of Cholesterol Hydroxylase in the Liver of Mice

**DOI:** 10.3389/fnut.2021.761928

**Published:** 2021-10-27

**Authors:** Koki Sugimoto, Ryota Hosomi, Munehiro Yoshida, Kenji Fukunaga

**Affiliations:** Laboratory of Food and Nutritional Sciences, Faculty of Chemistry, Materials and Bioengineering, Kansai University, Suita, Japan

**Keywords:** Japanese giant scallop (*Patinopecten yessoensis*), phospholipids, *n*-3 polyunsaturated fatty acids, eicosapentaenoic acid, cholesterol metabolism

## Abstract

In this study, we successfully prepared scallop oil (SCO), which contains high levels of phospholipids (PL) and eicosapentaenoic acid (EPA), from the internal organs of the Japanese giant scallop (*Patinopecten yessoensis*), one of the most important underutilized fishery resources in Japan. The intake of SCO lowers the serum and liver cholesterol contents in mice; however, whether the fatty acids (FA) composition or PL of SCO exhibits any cholesterol-lowering effect remains unknown. To elucidate whether the cholesterol-lowering function is due to FA composition or PL of SCO, and investigate the cholesterol-lowering mechanism by SCO, in the present study, mice were fed SCO's PL fraction (SCO-PL), triglyceride (TG)-type oil with almost the same FA composition as SCO-PL, called SCO's TG fraction (SCO-TG), soybean oil (SOY-TG), and soybean's PL fraction (SOY-PL). Male C57BL/6J mice (5-week-old) were fed high-fat and cholesterol diets containing 3% (w/w) experimental oils (SOY-TG, SOY-PL, SCO-TG, and SCO-PL) for 28 days. The SCO-PL diet significantly decreased the serum and liver cholesterol contents compared with the SOY-TG diet, but the intake of SOY-PL and SCO-TG did not show this effect. This result indicated that the serum and liver cholesterol-lowering effect observed in the SCO intake group was due to the effect of SCO-PL. The cholesterol-lowering effect of SCO-PL was in part related to the promotion of liver cholesterol 7α-hydroxylase (CYP7A1) expression, which is the rate-limiting enzyme for bile acid synthesis. In contrast, the expression levels of the ileum farnesoid X receptor (*Fxr*) and fibroblast growth factor 15 (*Fgf15*), which inhibit the expression of liver CYP7A1, were significantly reduced in the SCO-PL group than the SOY-TG group. From these results, the increase in the liver CYP7A1 expression by dietary SCO-PL was in part through the reduction of the ileum *Fxr/Fgf15* regulatory pathway. Therefore, this study showed that SCO-PL may be a health-promoting component as it lowers the serum and liver cholesterol contents by increasing the liver CYP7A1 expression, which is not seen in SOY-PL and SCO-TG.

## Introduction

Atherosclerosis is the main cause of cardiovascular disease (CVD), which is associated with high morbidity and mortality worldwide (1) and is characterized by cholesterol accumulation in the arterial walls and the development of lesions (2). High cholesterol and saturated fatty acids (FA) intake have been reported to cause atherosclerosis in animals and humans (3–7). Furthermore, excessive intake of these lipids is thought to promote atheroprogression through hypercholesterolemia, inflammation, and dysbiosis (8–10). Therefore, to prevent atherosclerosis, one of the most important ways to prevent atherosclerosis is to improve hypercholesterolemia caused by excessive intake of cholesterol and saturated FA.

Some edible oils have been reported to prevent hypercholesterolemia and atherosclerosis (11–20). Dietary eicosapentaenoic acid (EPA) and docosahexaenoic acid (DHA), which are *n*-3 polyunsaturated fatty acids (PUFA), reduce the incidence and mortality of arteriosclerosis *via* multiple mechanisms, including the decreased of serum triglyceride (TG) contents, antiplatelet aggregability, and antiarrhythmic effects (12). In contrast, dietary phospholipids (PL) have been demonstrated to decrease the serum total cholesterol and low-density lipoprotein cholesterol levels, and change total/high-density lipoprotein-cholesterol (HDL-C) in humans (13, 14). In addition, supplementation of polar lipids from gilthead sea bream (*Sparus aurata*) inhibits early atherosclerosis development in diet induced hypercholesterolemic through regulation of platelet activating factor metabolism in rabbit (15). PL intake has been shown to inhibit the cholesterol absorption in intestinal epithelial cells in animals and humans studies (11, 16, 17). Egg PL, which contains phosphatidylcholine (PtdCho) and sphingomyelin (CerPCho), are thought to inhibit the absorption of cholesterol and FA by inhibiting the mobilization of lipids from mixed micelles (18, 19). Cholesterol absorption is widely recognized to influence serum lipid contents (20). Thus, the inhibition of cholesterol absorption in the small intestine by PL intake is an attractive target for decreasing serum cholesterol contents and reducing the risk of atherosclerosis development. Our previous report showed that dietary PL containing *n*-3 PUFA decreased the serum cholesterol contents compared to TG containing *n*-3 PUFA (21). Consequently, *n*-3 PUFA and PL have attracted attention as supplement and functional food materials to prevent hypercholesterolemia and atherosclerosis.

The internal organs of the Japanese giant scallop (*Patinopecten yessoensis*) is a significant underutilized Japan's fishery resource, which contains a large amount of *n*-3 PUFA (22, 23). However, this has not been utilized effectively due to the presence of cadmium and diarrhetic shellfish poison (24). By removing the cadmium and diarrhetic shellfish poison from the internal organs of scallop, we have successfully prepared scallop oil (SCO) that satisfies the specifications for utilization as food. SCO safety was confirmed by the bacterial reverse mutation test, a micronucleus test (25), and studies of single and repeated doses in rodents (26, 27). In addition, SCO contains approximately 20 wt% of PL and includes higher EPA than standard TG-type fish oil (26). Our previous study showed that SCO intake lowered the serum and liver cholesterol contents in mice (28), and this effect was not observed in krill oil and menhaden oil intake (29). However, it is unclear whether the FA composition or PL of SCO has a cholesterol-lowering effect. To elucidate whether the cholesterol-lowering effect is due to the FA composition or PL of SCO and the cholesterol-lowering mechanism of SCO, C57BL/6J mice were fed SCO's PL fraction (SCO-PL), TG-type oil with almost the same FA composition as SCO-PL, called SCO's TG fraction (SCO-TG), soybean oil (SOY-TG), and soybean's PL fraction (SOY-PL) in high-fat and cholesterol-containing diets. Since it has been reported that female hormones affect blood cholesterol levels, male mice were used in this experiment (30).

## Materials and Methods

### Materials

SCO was prepared from the scallop internal organs, which were collected between August and September 2017, according to our previous report (25). SOY-PL and SCO-PL were obtained by dissolving soybean lecithin (Kanto Chemical Co., Inc., Tokyo, Japan) and SCO in cold acetone and collecting an insoluble fraction (31). Lard and SOY-TG were purchased from Junsei Chemical Co., Ltd. (Tokyo, Japan) and Merck KGaA (Darmstadt, Germany), respectively. The ingredients for the experimental diet were obtained from Oriental Yeast Co., Ltd. (Tokyo, Japan) and Fujifilm Wako Pure Chemical Co. (Osaka, Japan). Other chemicals were purchased from Merck KGaA, Tokyo Chemical Industry Co., Ltd. (Tokyo, Japan), and Nacalai Tesque, Inc. (Kyoto, Japan).

### Lipid Analysis of the Experimental Oils and Diets

After methylation with a boron trifluoride methanol complex solution, the FA compositions of the experimental oils and diets were analyzed using a gas chromatography (GC) system (GC-2014; Shimadzu Co., Kyoto, Japan) equipped with an Omegawax® capillary GC column (cat no. 24152; Merck KGaA) (32). After saponification with sodium hydroxide and 5α-cholestane was used as an internal standard, the cholesterol contents of the experimental oils were analyzed using a GC system equipped with an SH-Rtx-5MS column (cat no. 221-75701-30; Shimadzu GLC Ltd., Tokyo, Japan) (33). The PL contents of the experimental oils were measured using a phosphorus assay (34). The PL class compositions of SOY-PL and SCO-PL were analyzed by thin-layer chromatography using authentic PL standards, ceramide aminoethyl phosphate (CAEP), phosphatidic acid (PtdOH), PtdCho, phosphatidylethanolamine (PtdEtn), and phosphatidylinositol (PtdIns), according to our previous report (29). The glycerophospholipid (GPL) subclass composition of the experimental oils was analyzed based on the methods described by Dawson (35), with some modifications. Briefly, SOY-PL and SCO-PL were saponified with 0.5 M KOH at 75°C for 30 min and separated into two layers with water and chloroform. The upper layer was used as the diacyl-type PL fraction. The lower layer was heated at 75°C for 2 h with 2 M HCl-methanol and divided into two layers with water and chloroform. The upper layer was used as the plasmalogen (Pls) fraction, and the lower layer was used as an alkyl-acyl type PL. Each PL content was determined using a phosphorus assay (34), and the PL subclass compositions of the experimental oils were calculated from the phosphorus ratio.

### Animal Diet and Care

Male C57BL/6J mice (4-week-old) were obtained from Japan SLC Inc. (Shizuoka, Japan). After an acclimatization period of 7 days, we divided the mice into four groups of eight mice each, so that their average body weight (BW) would be similar. The mice were bred in an air-conditioned room (light on, 8:00–20:00; temperature, 20–22°C) with free access to drinking water. Mice in the SOY-TG group were given the American Institute of Nutrition 93G formula (36) modified high-fat diet [22% (w/w) lard, 8% (w/w) SOY-TG, 0.5% (w/w) cholesterol, and 0.1% (w/w) cholic acid]. Mice in the SOY-PL, SCO-TG, and SCO-PL groups were fed diets in which SOY-TG was replaced by about 3% (w/w) of each the experimental oils, respectively, to unify the energy ratio obtained from fat. The SOY-PL and SCO-PL diets were prepared to contain 3% PL, and the SCO-TG diet was prepared with approximately the same *n*-3 PUFA content as the SCO-PL diet. Additionally, the experimental diet was adjusted to 0.5% cholesterol by adding cholesterol. The ingredients of the experimental diets are listed in Supplementary Table 1. New diets were provided every day by pair-feeding. BW was measured daily. The feces of each mouse were collected daily for 2 days before sacrifice, and then weighed, frozen, and ground using a conventional mill. After 28 days of experimental diet administration, the mice that did not fast were anesthetized with isoflurane (Fujifilm Wako Pure Chemical Co.) and then sacrificed (9:00–12:00). Blood was collected, and then serum was obtained by centrifugation at 2,000 × *g* for 15 min. The organs including liver, jejunum, ileum, as well as white adipose tissue (WAT) from the epididymal, mesenteric, perirenal, and inguinal WAT were removed, rinsed with cold saline, and weighed. The organs were frozen in liquid nitrogen and stored at −80°C until analysis. A portion of the liver and mucosa of the jejunum and ileum were preserved in RNAlater® solution (Merck KGaA) for stable storage of RNA.

### Biochemical Analysis of Serum, Liver, and Feces

Serum lipid parameters including TG, PL, total cholesterol, HDL-C, and non-high-density lipoprotein cholesterol (non-HDL-C) contents were measured using an Olympus AU5431 (Olympus Co., Tokyo, Japan) by Japan Medical Laboratory (Kaizuka, Japan).

Liver total lipids were extracted using by Bligh and Dyer (37) method and then dissolved in 2-propanol. Following the manufacturer's instructions, liver TG content was then determined using the Triglyceride E-Test Wako (Fujifilm Wako Pure Chemical Co.). The liver PL and cholesterol contents were measured using the same methods described in “Lipid Analysis of the Experimental Oils and Diets” section. The liver BA composition was analyzed using GC-mass spectrometry (MS), as described in our previous report (29).

The fecal moisture content was measured by freeze-drying (FDU-1200; Tokyo Rikakikai Co. Ltd., Tokyo, Japan). The fecal neutral sterols, including cholesterol and coprostanol, were measured by GC using the same methods described in “Lipid Analysis of the Experimental Oils and Diets” section (31). Following the manufacturer's instructions, the fecal total bile acid (BA) content was analyzed using the Total Bile Acids Test Wako (Fujifilm Wako Pure Chemical Co.). The fecal total sterol content was sum of neutral sterol and total BA contents. The fecal BA composition was measured by GC-MS using the same methods as described above (29). Feces collected on the 27 and 28 days were used to measure water and neutral sterols contents, and BA composition and total BA content, respectively.

### mRNA Expression Analysis

RNA isolation and cDNA synthesis of the liver, jejunum, and ileum were conducted using the TRIzol^®^ reagent (Thermo Fisher Scientific Inc., Waltham, MA, USA) and GoScript^TM^ Reverse Transcription System (Promega Co., Madison, WI, USA), respectively. In addition, the mRNA expression levels were analyzed in duplicate by a Thermal Cycler Dice® Real Time System (Takara Bio Inc., Kusatsu, Japan) and GoTaq^®^ qPCR Master Mix (Promega Co.). The expression levels of the following genes were measured; adenosine tri-phosphate-binding cassette (*Abc*) *a1, Abcg5, Abcg8*, acetyl-Coenzyme A acetyltransferase 1 (*Acat1*), cytochrome P450 family 2 subfamily c polypeptide 70 (*Cyp2c70*), cytochrome P450 family 7 subfamily a polypeptide 1 (*Cyp7a1*), cytochrome P450 family 7 subfamily b polypeptide 1 (*Cyp7b1*), cytochrome P450 family 8 subfamily b polypeptide 1 (*Cyp8b1*), cytochrome P450 family 27 subfamily a polypeptide 1 (*Cyp27a1*), fibroblast growth factor 15 (*Fgf15*), fibroblast growth factor receptor 4 (*Fgfr4*), farnesoid X receptor (*Fxr*), 3-hydroxy-3-methylglutaryl coenzyme A reductase (*Hmgcr*), ileal bile acid transporter (*Ibat*), low density lipoprotein receptor (*Ldlr*), liver receptor homolog 1 (*Lrh1*), liver X receptor (*Lxr*), niemann-pick C1 like 1 (*Npc1l1*), small heterodimer partner 1 (*Shp1*), scavenger receptor class B type 1 (*Srb1*), sterol regulatory element binding factor 2 (*Srebf2*), and glyceraldehyde 3-phosphate dehydrogenase (*Gapdh*). The primer sequence was designed using Primer3Plus (http://primer3plus.com/), and are listed in Supplementary Table 2. The mRNA expression levels were normalized to the *Gapdh* levels and expressed as the fold-change in mRNA expression relative to the SOY-TG group.

### Western Blotting Analysis

The liver tissue was homogenized with a bead beater-type homogenizer in 10 volumes of 3 mM Tris-hydrogen chloride buffer (pH 7.4) containing 0.25 M sucrose, 1 mM ethylenediaminetetraacetic acid, and the protease inhibitor cocktail (Merck KGaA). After centrifugation (500 × *g* at 4°C for 10 min), the supernatant was used for western blotting analyses of CYP7A1 and GAPDH. First, the total protein content was determined using the protein assay BCA kit (Nacalai Tesque, Inc.). After the total protein (liver 5 mg of protein/lane) was separated by sodium dodecyl sulfate-polyacrylamide gel electrophoresis (38), the separated proteins were transferred to a polyvinylidene fluoride membrane. Then, CYP7A1 and GAPDH expression levels were detected using a specific primary antibody (cat. no. sc-518007 and sc-32233; Santa Cruz Biotechnology Inc., Dellas, TX, USA), a horseradish peroxidase-conjugated secondary antibody (cat no. sc-516102, Santa Cruz Biotechnology Inc.), and chemiluminescent substrate solutions (ATTO Corporation, Tokyo, Japan), and detection of the band with ImageQuant LAS 500 (Cytiva, Tokyo, Japan) according to the manufacturer's instructions. The relative densities of each band were quantitatively determined using ImageQuant TL software (Cytiva) and normalized to GAPDH.

### Statistical Analysis

The data are expressed as the mean ± standard error of the mean (SEM) and assessed by one-way analysis of variance. And then Tukey's multiple comparison test was conducted to determine the differences between multiple groups (*p* < 0.05). These statistical tests were performed using statistical program package the GraphPad Prism8 software for Mac (GraphPad Software, San Diego, CA, USA).

## Results

### Experimental Oils Composition

The lipid compositions of the experimental oils are listed in Table 1. SCO-TG and SCO-PL contained both 77.0 mg/g of EPA, 2.8 and 2.9 mg/g of docosapentaenoic acid, and 62.3 and 62.1 mg/g of DHA as *n*-3 PUFA, respectively. On the other hand, SOY-TG and SOY-PL contained 63.3 and 34.6 mg/g of α-linolenic acid (C18:3*n*-3) as *n*-3 PUFA, respectively. Among the experimental oils, only SCO-PL contained 0.5 mg/g of cholesterol. In addition, SOY-PL and SCO-PL contained 828 and 889 mg/g of PL, respectively.

**Table 1:** Lipid profile of the experimental oils.

	**Experimental oils**				
	**SOY-TG**	**SOY-PL**	**SCO-TG**	**SCO-PL**	**Lard**
**Fatty acid composition (mg/g)**					
C14:0	0.6	0.4	32.7	5.4	15.0
C16:0	99.4	104.8	80.1	41.7	230.4
C16:1*n*-7	0.8	0.6	6.0	6.9	27.2
C18:0	37.4	22.5	24.2	27.2	125.8
C18:1*n*-9	229.5	46.7	527.5	5.5	413.6
C18:1*n*-7	12.5	7.0	15.2	12.5	29.1
C18:2*n*-6	512.8	328.6	61.9	N.D.	64.8
C18:3*n*-3	63.3	34.6	4.9	N.D.	3.2
C20:1*n*-9	0.5	N.D.	6.0	16.3	6.4
C20:4*n*-6	N.D.	N.D.	4.5	19.6	N.D.
C20:5*n*-3 (EPA)	N.D.	N.D.	77.0	77.0	N.D.
C22:5*n*-3	N.D.	N.D.	2.8	2.9	N.D.
C22:6*n*-3 (DHA)	N.D.	N.D.	62.3	62.1	N.D.
Others	8.4	7.9	43.0	24.7	12.5
**PL and cholesterol contents (mg/g)**					
PL (mg/g)	N.D.	828	N.D.	888	N.D.
Cholesterol (mg/g)	N.D.	N.D.	N.D.	0.5	N.D.

*DHA, docosahexaenoic acid; EPA, eicosapentaenoic acid; N.D., not detected; PL, phospholipids; SCO-PL, scallop oil's phospholipids fraction; SCO-TG, scallop oil's triglyceride fraction; SOY-PL, soybean oil's phospholipids fraction; SOY-TG, soybean oil*.

The PL class composition and subclass composition of SOY-PL and SCO-PL are listed in Table 2. The PL class composition of SOY-PL was 36.6 wt% of PtdCho, 30.8 wt% of PtdEtn, 20.5 wt% of PtdIns, and 6.3 wt% of PtdOH, and that of SCO-PL was 63.4 wt% of PtdCho, 21.1 wt% of PtdEtn, and 8.7 wt% of CAEP. The GPL subclass composition of SOY-PL was 99.4 mol% of diacyl-type and 0.6 mol% of alkyl-acyl type, and that of SCO-PL was 89.9 mol% of diacyl type, 6.4 mol% of Pls, and 3.7 mol% of alkyl-acyl type.

**Table 2:** Phospholipids class and subclass composition of the experimental oils.

	**Experimental oils**	
	**SOY-PL**	**SCO-PL**
**PL class composition (wt%)**		
PtdCho	36.6	63.4
PtdEtn	30.8	21.1
PtdIns	20.5	N.D.
PtdOH	6.3	N.D.
CAEP	N.D.	8.7
Others	5.9	6.7
**GPL subclass composition (mol%)**		
Diacyl type	99.4	89.9
Pls	N.D.	6.4
Alkyl-acyl type	0.6	3.7

*CAEP, ceramide aminoethyl phosphate; GPL, glycerophospholipids; N.D., not detected; PtdCho, phosphatidylcholine; PtdEtn, phosphatidylethanolamine; PtdIns, phosphatidylinositol; PtdOH, phosphatidic acid; PL, phospholipids; Pls, plasmalogen; SCO-PL, scallop oil's phospholipids fraction; SOY-PL, soybean oil's phospholipids fraction*.

The main FA compositions of the experimental diets are shown in Table 3. FA contained in all diets was mainly composed of palmitic acid (C16:0), stearic acid (C18:0), oleic acid (C18:1*n*-9), linoleic acid (C18:2*n*-6), and α-linolenic acid (C18:3*n*-3). The SCO-TG and SCO-PL diets contained almost the same amounts of EPA and DHA.

**Table 3:** Main fatty acid composition of the experimental diets.

	**Experimental groups**			
	**SOY-TG**	**SOY-PL**	**SCO-TG**	**SCO-PL**
	**mg/g**			
C16:0	58.6	58.8	58.0	56.7
C18:0	30.7	30.1	30.2	30.3
C18:1*n*-9	109.4	102.7	119.4	101.8
C18:2*n*-6	55.3	48.6	40.1	38.0
C18:3*n*-3	5.8	4.7	3.8	3.6
C20:5*n*-3 (EPA)	N.D.	N.D.	2.6	2.6
C22:6*n*-3 (DHA)	N.D.	N.D.	2.1	2.1

*DHA, docosahexaenoic acid; EPA, eicosapentaenoic acid; N.D., not detected; SCO-PL, scallop oil's phospholipids fraction; SCO-TG, scallop oil's triglyceride fraction; SOY-PL, soybean oil's phospholipids fraction; SOY-TG, soybean oil*.

### Growth Parameters and Relative Organ Weights

Growth parameters during the feeding period of 28 days and relative organ weights are shown in Table 4. There were no significant differences in the initial BW, final BW, BW gain, and food intake among the groups. However, the SCO-PL group was significantly lower the relative liver weight than the SOY-TG group. The relative WAT (epididymal, mesenteric, perirenal, and inguinal WAT) weights were not significantly different among the groups.

**Table 4:** Growth parameters and relative organ weights.

	**Experimental groups**			
	**SOY-TG**	**SOY-PL**	**SCO-TG**	**SCO-PL**
**Growth parameters**				
Initial BW (g)	19.9 ± 0.4	19.9 ± 0.3	19.9 ± 0.3	20.0 ± 0.2
Final BW (g)	23.8 ± 0.5	24.5 ± 0.5	24.0 ± 0.4	23.1 ± 0.4
BW gain (g/day)	0.14 ± 0.02	0.17 ± 0.01	0.15 ± 0.01	0.11 ± 0.02
Food intake (g/day)	2.3 ± 0.1	2.2 ± 0.1	2.2 ± 0.0	2.3 ± 0.0
**Relative organ weight (g/100g BW)**				
Liver	4.54 ± 0.23^b^	4.42 ± 0.10^ab^	4.49 ± 0.11^ab^	3.76 ± 0.26^a^
Epididymal WAT	3.01 ± 0.31	3.17 ± 0.22	3.35 ± 0.17	2.84 ± 0.22
Mesenteric WAT	1.32 ± 0.08	1.37 ± 0.04	1.29 ± 0.06	1.30 ± 0.07
Perirenal WAT	0.97 ± 0.15	1.00 ± 0.07	0.92 ± 0.10	0.64 ± 0.10
Inguinal WAT	1.34 ± 0.14	1.44 ± 0.08	1.56 ± 0.12	1.21 ± 0.11

*Data represent the mean ± SEM (n = 8). Values in the same row not sharing a common superscript are significantly different (p < 0.05, Tukey's multiple comparison test)*.

*BW, body weight; SCO-PL, scallop oil's phospholipids fraction; SCO-TG, scallop oil's triglyceride fraction; SOY-PL, soybean oil's phospholipids fraction; SOY-TG, soybean oil; WAT, white adipose tissue*.

### Serum and Liver Lipid Contents

The serum and liver lipid contents are shown in Table 5. The SCO-PL group was significantly lower serum TG, PL, and HDL-C contents than the SOY-PL group. Mice fed the SCO-PL diet had significantly reduced serum total cholesterol content compared to mice fed the SOY-TG and SOY-PL diets. In addition, the SOY-TG group was significantly higher the serum non-HDL-C content than the other groups. The SOY-PL and SCO-PL diets significantly decreased the liver TG content compared to the SOY-TG diet. Compared to the SOY-TG and SCO-TG groups, the SCO-PL group had significantly lower liver cholesterol content.

**Table 5:** Lipid contents in the serum and liver.

	**Experimental groups**			
	**SOY-TG**	**SOY-PL**	**SCO-TG**	**SCO-PL**
**Serum (mg/dL)**				
TG	27 ±4^ab^	55 ± 10^b^	32 ± 4^ab^	25 ± 8^a^
PL	216 ± 19^ab^	274 ± 7^b^	220 ± 8^ab^	182 ± 25^a^
Total cholesterol	142 ± 9^b^	144 ± 4^b^	130 ± 5^ab^	110 ± 13^a^
HDL-C	90 ± 12^ab^	119 ± 3^b^	101 ± 3^ab^	86 ± 10^a^
Non-HDL-C	52 ± 13^b^	26 ± 2^a^	29 ± 2^a^	24 ± 3^a^
**Liver (mg/g)**				
TG	74.7 ± 6.9^b^	49.2 ± 4.2^a^	58.1 ± 6.4^ab^	46.9 ± 7.8^a^
PL	18.4 ± 1.3	19.5± 0.5	21.3 ± 0.5	21.3 ± 1.1
Cholesterol	20.4 ± 3.0^bc^	10.8 ± 1.2^ab^	25.3 ± 4.2^c^	9.0 ± 1.2^a^

*Data represent the mean ± SEM (n = 8). Values in the same row not sharing a common superscript are significantly different (p < 0.05, Tukey's multiple comparison test)*.

*HDL-C, high-density lipoprotein cholesterol; Non-HDL-C, non-high-density lipoprotein cholesterol; PL, phospholipids; SCO-PL, scallop oil's phospholipids fraction; SCO-TG, scallop oil's triglyceride fraction; SOY-PL, soybean oil's phospholipids fraction; SOY-TG, soybean oil; TG, triglyceride*.

**Figure 1:**
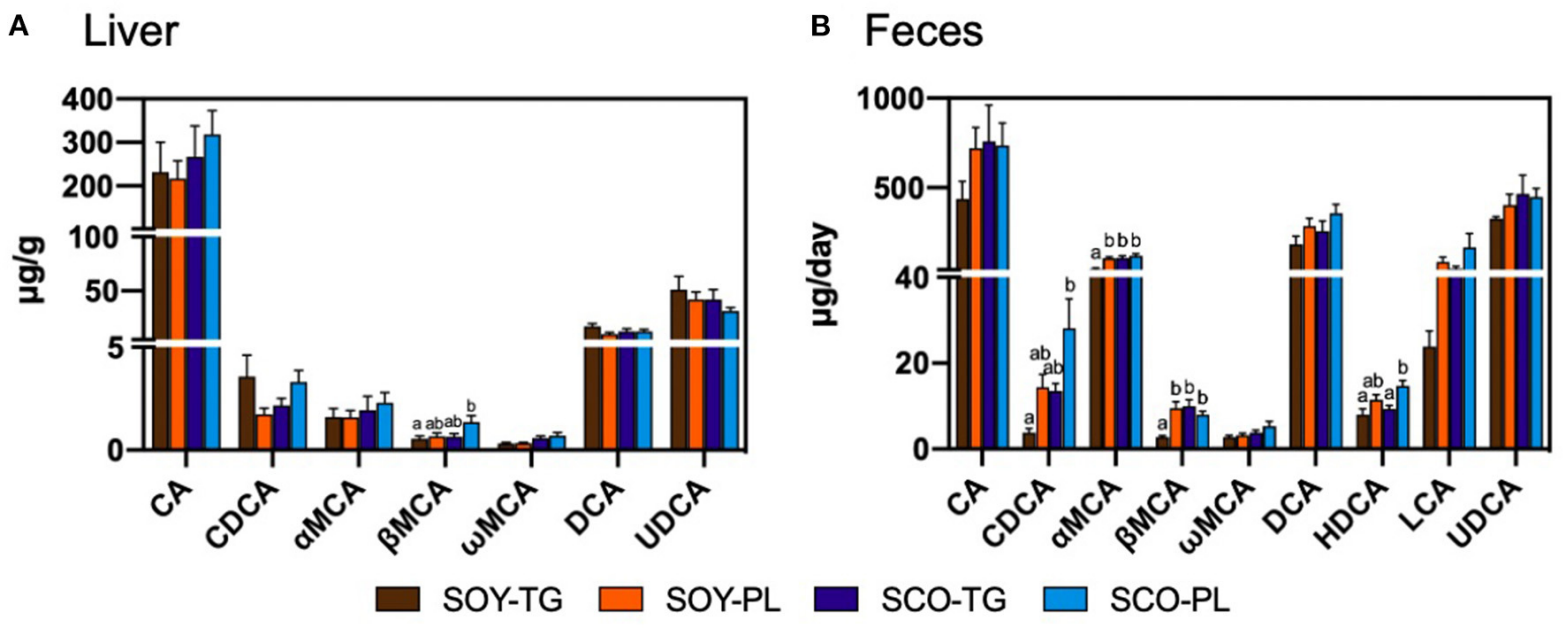
Bile acids composition in the liver **(A)** and feces **(B)**. Data represent the mean ± SEM (*n* = 8). Different letters indicate significantly different at *p* < 0.05 (Tukey's multiple comparison test). CA, cholic acid; CDCA, chenodeoxycholic acid; DCA, deoxycholic acid; HDCA, hyodeoxycholic acid; LCA, lithocholic acid; αMCA, α-muricholic acid; βMCA, β-muricholic acid; ωMCA, ω-muricholic acid; SCO-PL, scallop oil's phospholipids fraction; SCO-TG, scallop oil's triglyceride fraction; SOY-PL, soybean oil's phospholipids fraction; SOY-TG, soybean oil; UDCA, ursodeoxycholic acid.

Liver BA content is shown in Figure 1A. The SCO-PL group was significantly higher the liver β-muricholic acid (MCA) content than the SOY-TG group. In contrast, no significant differences were observed in the other BA contents in the liver among the groups.

### Fecal Moisture and Sterol Contents

The fecal moisture, neutral sterol, total BA, and total sterol contents are shown in Table 6. The fecal moisture content was not significantly different among the groups. Fecal neutral sterol was significantly increased in the SOY-PL, SCO-TG, and SCO-PL groups compared with the SOY-TG group, in which the cholesterol content was significantly increased in the SCO-TG and SCO-PL groups compared with the SOY-TG and SOY-PL groups, and the coprostanol content was significantly increased in the SOY-PL group compared with the other groups. The SCO-PL group was significantly higher fecal total BA content than the SOY-TG and SCO-TG groups. In addition, the SOY-PL, SCO-TG, and SCO-PL diets increased the fecal total sterol content compared to the SOY-TG diet.

**Table 6:** Moisture and sterol contents excretions of the feces.

	**Experimental groups**			
	**SOY-TG**	**SOY-PL**	**SCO-TG**	**SCO-PL**
Moisture (wt%)	24.5 ± 1.4	23.4 ± 2.4	21.1 ± 1.6	22.6 ± 3.8
Neutral sterol (mg/day)^1^	5.9 ± 0.5^a^	12.4 ± 0.3^b^	14.4 ± 0.7^b^	13.5 ± 0.5^b^
Cholesterol	5.6 ± 0.4^a^	7.0 ± 0.7^a^	13.8 ± 0.7^b^	13.3 ± 0.5^b^
Coprostanol	0.3 ± 0.1^a^	5.4 ± 0.8^b^	0.6 ± 0.1^a^	0.2 ± 0.0^a^
Total BA (mg/day)	1.5 ± 0.1^a^	1.6 ± 0.1^ab^	1.5 ± 0.1^a^	1.9 ± 0.1^b^
Total sterol (mg/day)^2^	7.4 ± 0.5^a^	13.9 ± 0.3^b^	15.8 ± 0.6^b^	15.4 ± 0.5^b^

*Data represent the mean ± SEM (n = 8). Values in the same row not sharing a common superscript are significantly different (p < 0.05, Tukey's multiple comparison test)*.

1 *Neutral sterol is the sum of cholesterol and coprostanol*.

2 *Total sterol is the sum of neutral sterols and total bile acids*.

*BA, bile acids; SCO-PL, scallop oil's phospholipids fraction; SCO-TG, scallop oil's triglyceride fraction; SOY-PL, soybean oil's phospholipids fraction; SOY-TG, soybean oil*.

Fecal BA content is shown in Figure 1B. The SCO-PL group was significantly higher the fecal chenodeoxycholic acid (CDCA) content than the SOY-TG groups and the fecal hyodeoxycholic acid (HDCA) content than the SOY-TG and SCO-TG groups. The SOY-TG group was significantly lower fecal αMCA and βMCA contents than the other groups. There were no significant differences in the fecal CA, ωMCA, DCA, LCA, and UDCA among the groups.

### Relative mRNA and Protein Expression Levels of the Liver, Jejunum, and Ileum

**Figure 2:**
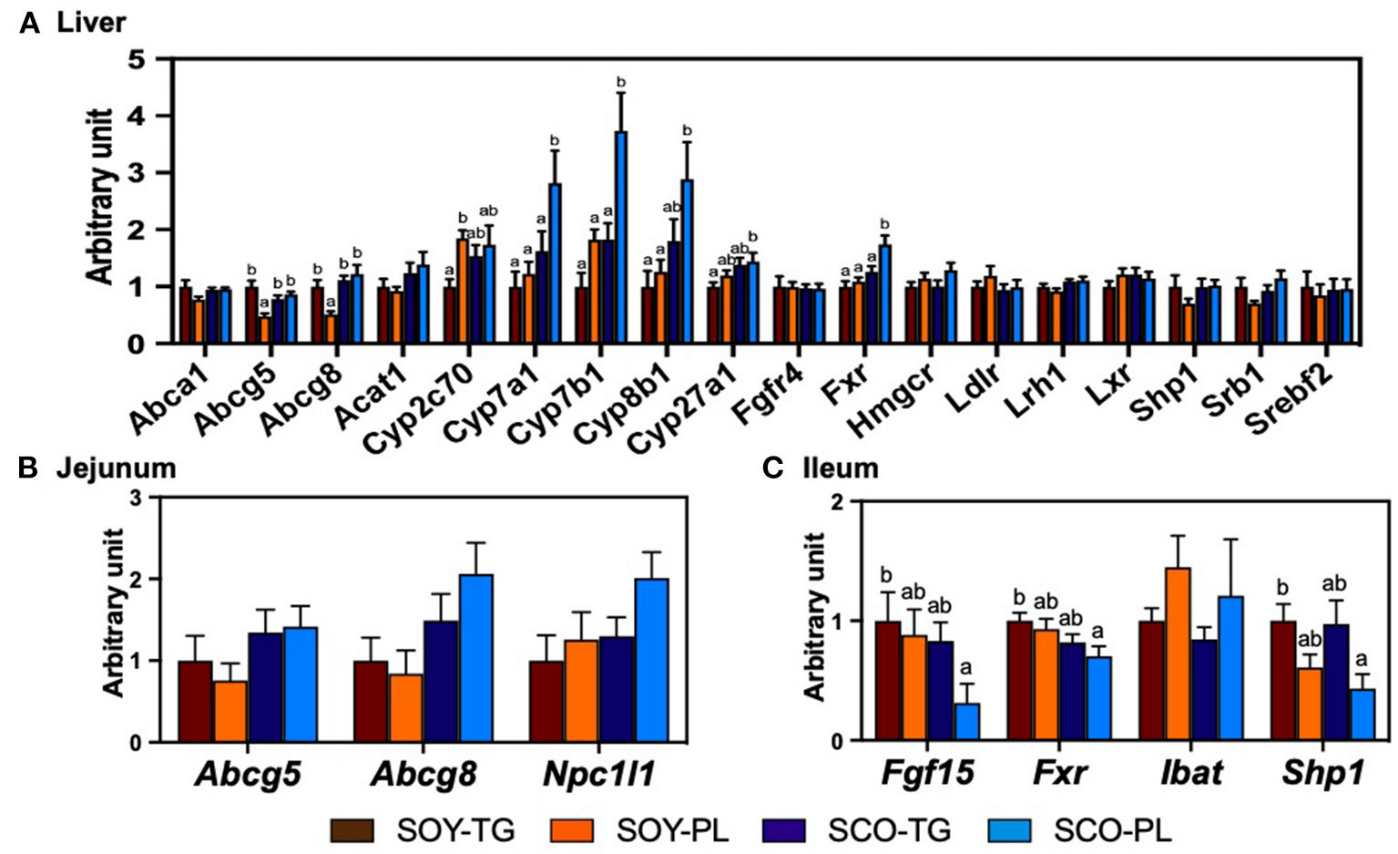
The mRNA expression levels of genes involved in cholesterol metabolism in the liver **(A)** and the mucosa of jejunum **(B)** and ileum **(C)**. Data represent the mean ± SEM (*n* = 8). Different letters indicate significantly different at *p* < 0.05 (Tukey's multiple comparison test). The mRNA expression levels were determined using the glyceraldehyde 3-phosphate dehydrogenase (*Gapdh*) expression levels for normalization. The mRNA expression levels of genes are shown relative to those determined from the livers of mice in the control group (set at 1). The abbreviation names of these genes are listed in Supplementary Table 2. SCO-PL, scallop oil's phospholipids fraction; SCO-TG, scallop oil's triglyceride fraction; SOY-PL, soybean oil's phospholipids fraction; SOY-TG, soybean oil.

The relative mRNA expression levels in the liver (Figure 2A), jejunum (Figure 2B), and ileum (Figure 2C) related to cholesterol metabolism are shown in Figure 2. The SCO-PL diet significantly increased the liver expression levels of *Cyp7a1, Cyp7b1*, and *Fxr* compared to the other diets, *Cyp27b1* compared to the SOY-TG diet, and *Cyp8b1* compared to the SOY-TG and SOY-PL diets. Liver *Abcg5* and *Abcg8* expression levels in the SOY-PL group were significantly decreased compared to those in the other groups. In addition, the SOY-PL diet significantly increased the liver *Cyp2c70* expression level compared to the SOY-TG group. The SCO-PL diet significantly decreased the ileum *Fgf15, Fxr*, and *Shp1* expression levels compared to the SOY-TG diet. In contrast, no significant differences were observed in jejunum *Abcg5, Abcg 8*, and *Npc1l1* expression levels among the groups.

**Figure 3:**
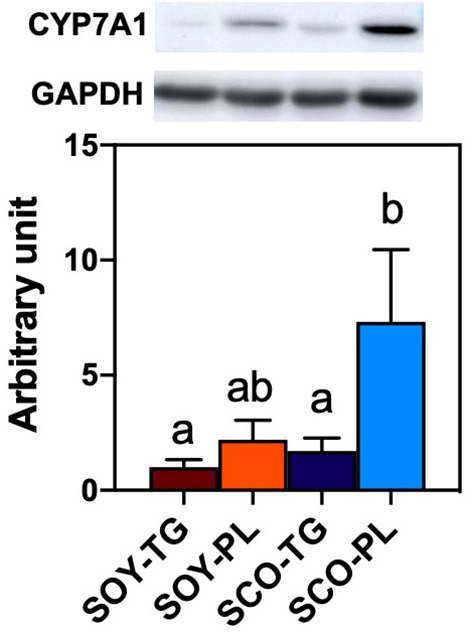
Liver CYP7A1 protein expression level. Data represent the mean ± SEM (*n* = 8). Different letters indicate significantly different at *p* < 0.05 (Tukey's multiple comparison test). The protein expression level was determined using the GAPDH expression level for normalization. CYP7A1 protein expression levels are shown relative to those determined from the livers of mice in the SOY-TG group (set at 1). The bands of representative 4 sample/group are shown in Supplementary Figure 1. CYP7A1, cytochrome P450 family 7 subfamily a polypeptide 1; GAPDH, glyceraldehyde 3-phosphate dehydrogenase; SCO-PL, scallop oil's phospholipids fraction; SCO-TG, scallop oil's triglyceride fraction; SOY-PL, soybean oil's phospholipids fraction; SOY-TG, soybean oil.

Relative liver CYP7A1 expression level is shown in Figure 3. Liver CYP7A1, which encodes the rate-limiting enzyme in the classical BA biosynthetic pathway, expression level in the SCO-PL group was significantly higher than that in the SOY-TG and SCO-TG groups.

## Discussion

Our previous study demonstrated that SCO intake decreased the serum and liver cholesterol content compared with *n*-3 PUFA-containing oils, including tuna oil, menhaden oil, and krill oil (28, 29). However, it is unclear whether the FA composition or PL of SCO is responsible for this cholesterol-lowering effect. In this study, the serum and liver cholesterol contents decreased in the SCO-PL group compared to those in the SOY-TG group, whereas the SCO-TG diet, which was prepared with almost the same FA content as the SCO-PL diet, did not decrease the cholesterol content (Table 5). This result indicated that the decrease in serum and liver cholesterol contents observed in the case of SCO intake is due to the effect of SCO-PL.

Several mechanisms could explain the effect of SCO-PL intake on lowering liver cholesterol content. Enhancement of fecal sterol excretion is the first possibility (21). The SOY-PL and SCO-PL diets significantly increased fecal neutral sterol excretion compared to the SOY-TG diet (Table 6). GPL, including PtdCho and PtdEtn, intake is known to reduce cholesterol absorption by inhibiting the hydrolysis of micellar PL (39, 40). CerPCho and sphingoid base formed hydrogen bonds with the hydroxyl group of cholesterol and inhibited the absorption of cholesterol (14, 41–43). No studies have reported the inhibition of cholesterol absorption by CAEP, a sphingolipid possessing a carbon-phosphorus bond, and is often found in bivalves. However, CAEP is hydrolyzed to sphingoid base during the digestive process and can interfere with cholesterol absorption (44). In this study, SOY-PL contained PtdCho and PtdEtn, and SCO-PL contained PtdCho, PtdEtn, and CAEP. These presences could have enhanced fecal neutral sterol excretion. In addition, fecal coprostanol excretion in the SOY-PL group was significantly increased compared to that in the other groups. Coprostanol is produced by hydrogenating cholesterol into intestinal bacteria, *Bacteroides, Clostridium*, and *Bifidobacterium* genera, and it is excreted into the feces with little absorption (45). Thus, SOY-PL intake might have increased fecal neutral sterol excretion due to the enhancement of coprostanol-producing bacteria. In contrast, the SCO-TG diet, which did not contain PL, increased neutral sterol excretion compared to the SOY-TG diet. Alvaro et al. (46) have shown that dietary *n*-3 PUFA changes the expression levels of the jejunum cholesterol transporters, *Abcg5* and *8* and *Npc1l1*, expression levels, and that it could lower cholesterol contents in the body. However, there were no differences in jejunum *Abcg5* and *8* and *Npc1l1* expression levels among the groups (Figure 2B). These cholesterol transporter gene expression levels may be affected by a high-fat diet containing cholesterol and cholic acid, and further research is needed to determine why SCO-TG intake increases fecal neutral sterol excretion. From these results, the enhancement of fecal sterol excretion by SCO-PL intake could cause serum and liver cholesterol-lowering effects, although the SOY-PL and SCO-TG groups also increased fecal sterol excretion.

The second possible cause of lowering cholesterol content is an alteration in liver cholesterol metabolism. The SCO-PL diet increased the mRNA expression levels of *Cyp7b1, Cyp8b1*, and *Cyp27a1* in the BA biosynthetic pathway (Figure 2A). Furthermore, liver CYP7A1 at mRNA and protein expression levels in the SCO-PL group was significantly increased compared to that in the other groups (Figure 3). Li et al. (47) reported that overexpression of CYP7A1 promoted hepatic BA synthesis and secretion into bile in mice. Moreover, Pandak et al. (48) showed that the overexpression of CYP7A1 in hepatocytes increased BA synthesis and lowered cholesterol content. Our previous study showed that SCO intake increases the mRNA expression levels of liver *Cyp7a1* (29). In this study, SCO-TG intake did not increase liver CYP7A1 expression; therefore, the increase in *Cyp7a1* expression observed SCO intake was due to the effect of SCO-PL.

Several pathways are known to control liver CYP7A1 expression, including the FXR/SHP1 pathway (49, 50), mitogen-activated protein kinase-c-Jun N-terminal kinase (JNK) pathway (51, 52), and the pregnane X receptor pathway (53). In addition, the ileum FXR is involved in cholesterol metabolism; for example, it regulates ileum FGF15 and SHP1 (54). It has been reported that ileum FGF15 expression is involved in liver CYP7A1 expression (55, 56). In detail, ileum FGF15 is transported from the portal vein to the liver and suppresses the transcription of CYP7A1 by phosphorylating JNK *via* FGFR4. Sayin et al. (57) have demonstrated a negative correlation between the expression levels of ileum *Fgf15* and liver *Cyp7a1* in mice. In this study, the expression levels of ileum *Fgf15* and *Shp1* were lower in the SCO-PL group (Figure 2C), and these reductions could be due to a decrease in the ileum *Fxr* expression level. Moreover, there was a negative correlation between ileum *Fgf15* and liver *Cyp7a1* expression levels (*r* = −0.42, *p* = 0.02, Supplementary Figure 2). These results showed that the enhancement of liver CYP7A1 expression in the SCO-PL group was partly due to the regulation of the ileum *Fgf15* expression level. Several BA species are recognized as regulators of cholesterol metabolism through ligand-activated transcription factors of FXR (58). For example, taurine-conjugated βMCA (TβMCA) has been reported to be an antagonist of ileum FXR (59). In mice, BA is usually taurine-conjugated in the liver (60, 61). In the present study, the SCO-PL diet significantly increased the liver and feces βMCA content (Figure 1B) and tended to increase the expression level of liver *Cyp2c70*, which synthesizes MCA from CDCA compared with the SOY-TG diet (*p* = 0.07, Figure 2A). Although the gallbladder BA composition could not be analyzed in this study, it has been reported that BA composition in the gallbladder is similar to that in the liver (60). Thus, the gallbladder and ileum βMCA content in the SCO-PL group might be higher than that in the SOY-TG group. From these results, the increase in liver CYP7A1 expression level by dietary SCO-PL in part through the reduction of the ileum expression level of *Fgf15* due to the increase in ileum TβMCA, which is an antagonist of FXR.

In this study, the cholesterol-lowering effect was observed in SCO-PL but not in SCO-TG, suggesting that the substances in SCO-PL have cholesterol-lowering effects. Both SOY-PL and SCO-PL intake increased fecal sterol excretion, but SOY-PL intake did not decrease serum and liver cholesterol contents. Therefore, the promotion of liver CYP7A1 expression observed only in the SCO-PL group could be highly associated with the cholesterol-lowering effect. SCO-PL consisted of 6.4 mol% of Pls, which was not present in SOY-PL (Table 2). Ding et al. (61) reported that a diet containing 1.0 wt% of EPA-enriched Pls improved cholesterol metabolism by enhancing the serum and gallbladder TβMCA contents. However, the SCO-PL diet contained 0.19 wt% Pls, and the EPA-bound form was even smaller in this study. Therefore, it is unlikely that Pls contained in SCO-PL affected the increase in liver CYP7A1 expression in mice. In addition, liver CYP7A1 expression levels were not enhanced by dietary SOY-PL, which contained PtdCho and PtdEtn. From these results, it is highly possible that the bioactive substance responsible for the enhancement of CYP7A1 expression is CAEP and alkyl-acyl type GPL, which are unique to SCO-PL. The upregulation of liver CYP7A1 expression by the intake of these substances is not yet known. In the future, the effects of CAEP and alkyl-acyl type GPL on the upregulation of liver CYP7A1 expression should be clarified.

We acknowledge there is a limitation in this study. The experimental group did not contain a no oil supplemented high-fat diet [22% (w/w) lard, 5% (w/w) SOY-TG, 0.5% (w/w) cholesterol, and 0.1% (w/w) cholic acid]. Since we are considering the application of SCO-PL as an ingredient for dietary supplements, it was necessary to compare the no oil supplemented high-fat diet group as a control. However, we concluded that the no oil supplemented high-fat diet was not appropriate as a control because the calorie ratios of carbohydrate, protein, and fat were different between the no oil supplemented high-fat and SCO-PL diets. In the future, it is necessary to clarify the cholesterol-lowering effect of SCO-PL in more detail by comparing it with a no oil supplemented high-fat diet.

## Conclusion

This study evaluated the effects of dietary SOY-TG, SOY-PL, SCO-TG, and SCO-PL on cholesterol metabolism in C57BL/6J mice fed a high-fat diet containing cholesterol. We found that SCO-PL intake lowered the serum and liver cholesterol contents compared to SOY-TG intake, but this effect was not observed with the intake of SOY-PL and SCO-TG. This effect was partly mediated by the enhancement of liver CYP7A1 expression levels and fecal total sterol excretion. In addition, the increase in liver CYP7A1 expression level by dietary SCO-PL was mediated partly by the reduction of the ileum *Fgf15* expression level. Thus, this study indicates that SCO-PL may be used as a health-promoting component to reduce the content of cholesterol in the body.

## Data Availability Statement

The original contributions presented in the study are included in the article/Supplementary Material, further inquiries can be directed to the corresponding author.

## Ethics Statement

The animal study was reviewed and approved by Animal Ethics Committee of Kansai University.

## Author Contributions

KS and RH performed the research, analyzed the data, and wrote the original manuscript. MY and KF supervised the research design and reviewed the manuscript. All authors contributed to and approved the final draft of the manuscript.

## Conflict of Interest

The authors declare that the research was conducted in the absence of any commercial or financial relationships that could be construed as a potential conflict of interest.

## Publisher's Note

All claims expressed in this article are solely those of the authors and do not necessarily represent those of their affiliated organizations, or those of the publisher, the editors and the reviewers. Any product that may be evaluated in this article, or claim that may be made by its manufacturer, is not guaranteed or endorsed by the publisher.
