# Supplementary material for: Dietary Phospholipids Prepared From Scallop Internal Organs Attenuate the Serum and Liver Cholesterol Contents by Enhancing the Expression of Cholesterol Hydroxylase in the Liver of Mice
Source: Front Nutr. 2021 Oct 27;8:761928. doi: 10.3389/fnut.2021.761928 (PMC8578998; doi:10.3389/fnut.2021.761928)
Supplement: Supplementary file 1 [file Data_Sheet_1.docx]

Supplementary Material

# Supplementary Figure


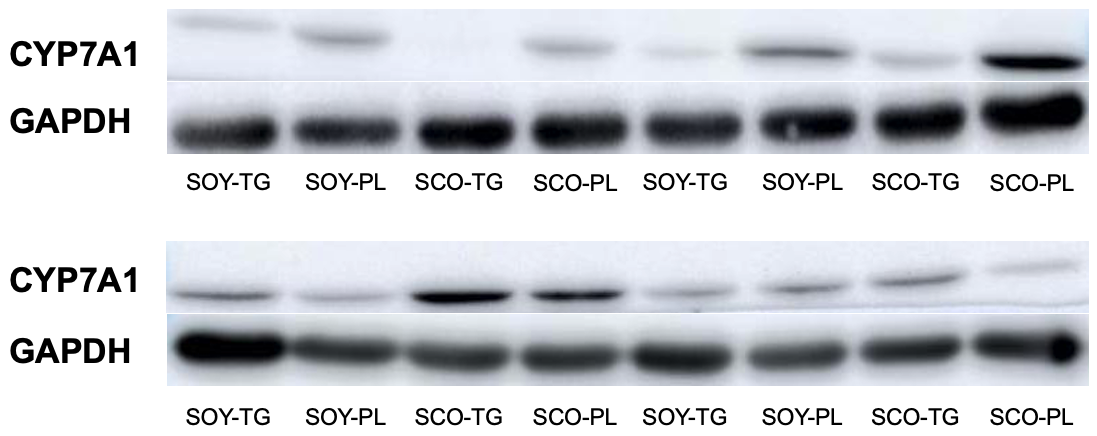


**Supplementary Figure 1**. Protein expression levels of CYP7A1 and GAPDH by western blot analysis.

Four representative data are shown for each group.

CYP7A1, cytochrome P450 family 7 subfamily a polypeptide 1; GAPDH, glyceraldehyde 3-phosphate dehydrogenase; SCO-PL, scallop oil’s phospholipid fraction; SCO-TG, scallop oil’s triglyceride fraction; SOY-PL, soybean oil’s phospholipid fraction; SOY-TG, soybean oil.

**
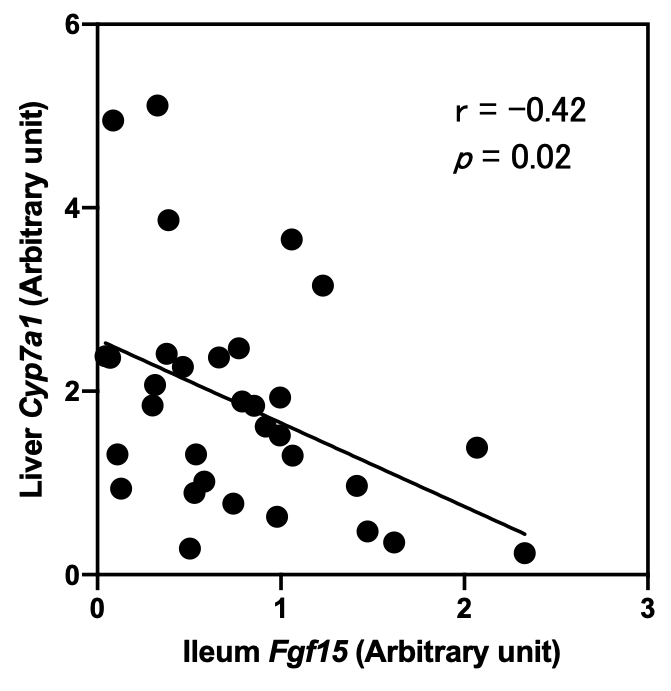
**

**Supplementary Figure 2.** Correlation between the expression levels of the ileum *Fgf15* and the liver *Cyp7a1*.

The data were evaluated using the Pearson’s correlation coefficient (n = 32). The mRNA expression levels were determined using the glyceraldehyde 3-phosphate dehydrogenase (*Gapdh*) expression levels for normalization.

*Cyp7a1*, cytochrome P450 family 7 subfamily a polypeptide 1; *Fgf15*, fibroblast growth factor 15.

# Supplementary Tables

**Supplementary Table 1.** Composition of the ingredients of the experimental diets.

| Ingredients | Experimental groups | | | |
| --- | --- | --- | --- | --- |
|  | SOY-TG | SOY-PL | SCO-TG | SCO-PL |
|  | g/kg | | | |
| Dextrinized corn starch | 60.2 | 60.2 | 60.2 | 60.2 |
| Corn starch | 175.286 | 175.286 | 175.286 | 175.306 |
| Casein | 258 | 258 | 258 | 258 |
| Sucrose | 100 | 100 | 100 | 100 |
| Cellulose | 50 | 50 | 50 | 50 |
| AIN-93G mineral mixture | 35 | 35 | 35 | 35 |
| AIN-93 vitamin mixture | 10 | 10 | 10 | 10 |
| L-Cystine | 3 | 3 | 3 | 3 |
| Choline bitartrate | 2.5 | 2.5 | 2.5 | 2.5 |
| *tert*-Butylhydroquinone | 0.014 | 0.014 | 0.014 | 0.014 |
| SOY-TG | 80 | 43.77 | 46.25 | 46.25 |
| SOY-PL |  | 36.23 |  |  |
| SCO-TG |  |  | 33.75 |  |
| SCO-PL |  |  |  | 33.75 |
| Lard | 220 | 220 | 220 | 220 |
| Cholesterol | 5 | 5 | 5 | 4.98 |
| Cholic acid | 1 | 1 | 1 | 1 |

SCO-PL, scallop oil’s phospholipids fraction; SCO-TG, scallop oil’s triglyceride fraction; SOY-PL, soybean oil’s phospholipids fraction; SOY-TG, soybean oil.

**Supplementary Table 2.** Primer sets used for real-time quantitative polymerase chain reaction (qPCR).

| Gene name |  | 5’→3’ primer sequence | Gene title |
| --- | --- | --- | --- |
| *Abca1* | Forward | AGTTTGTGGCCCTTTTGTGG | ATP-binding cassette A1 |
|  | Reverse | AAGACCAGGGCAATGCAAAC |  |
| *Abcg5* | Forward | TGCAGAGCGTTTTTCTG | ATP-binding cassette G5 |
|  | Reverse | TGTCATGACTGCCTCTACCTTC |  |
| *Abcg8* | Forward | ACGGTGGCAAAGACAAATCC | ATP-binding cassette G8 |
|  | Reverse | TGGCGTTTTGCTCTGTAAACG |  |
| *Acat1* | Forward | ATTTGCTGATGCTGCCGTAG | acyl-coenzyme A: cholesterol acyltransferase 1 |
|  | Reverse | AGCACAACCACACTGAATGC |  |
| *Cyp2c70* | Forward | AAACAAACCCGGCGTTTCTC | cytochrome P450 family 2 subfamily c polypeptide 70 |
|  | Reverse | GGGAGCCATTGGTTTTTCTCAG |  |
| *Cyp7a1* | Forward | TGGGCATCTCAAGCAAACAC | cytochrome P450 family 7 subfamily a polypeptide 1 |
|  | Reverse | TCAGAGGCTGCTTTCATTGC |  |
| *Cyp7b1* | Forward | AGTGTGGCAGAGAAGCTTTG | cytochrome P450 family 7 subfamily b polypeptide 1 |
|  | Reverse | TGTGACCCAAAACCAACTGG |  |
| *Cyp8b1* | Forward | TTGGTGATGCTAGGGCCTAAAG | cytochrome P450 family 8 subfamily b polypeptide 1 |
|  | Reverse | TGGTGTAGCCGAATAAGCTCAG |  |
| *Cyp27a1* | Forward | ACACGACATCCAACACACTG | cytochrome P450 family 27 subfamily a polypeptide 1 |
|  | Reverse | ATGTGGGCAAAGTCCTTGTG |  |
| *Fgf15* | Forward | ACCCTGTTGTGTTAGTGGCTAG | fibroblast growth factor 15 |
|  | Reverse | TATCAGCAGCCTCCAAAGTCAG |  |
| *Fgfr4* | Forward | ACAAAAGCTGTCCCGTTTCC | fibroblast growth factor receptor 4 |
|  | Reverse | AGGGACAAACTTGACTTGCC |  |
| *Fxr* | Forward | AGGGCTGCAAAGGTTTCTTC | farnesoid X receptor |
|  | Reverse | ACATGTCCATCACGCAGTTG |  |
| *Hmgcr* | Forward | TTGGTTTCTGGCGCTTTCAG | 3-hydroxy-3-methylglutaryl coenzyme A reductase |
|  | Reverse | AACACAGCACGGAAAGAACC |  |
| *Ibat* | Forward | AAGCATACTCAGCTGGCAGTAG | ileal bile acid transporter |
|  | Reverse | TGCTTGTGCTGTGCAAATGG |  |
| *Ldlr* | Forward | AATGGGGGCAATCGGAAAAC | low density lipoprotein receptor |
|  | Reverse | TGGCACTGAAAATGGCTTCG |  |

**Supplementary Table 2.** *continued.*

| Gene name |  | 5’→3’ primer sequence | Gene title |
| --- | --- | --- | --- |
| *Lrh1* | Forward | GCCTCAAGTTCAAGCGAAGATC | liver receptor homolog 1 |
|  | Reverse | AAAAGCCCAAATGCGCTCAG |  |
| *Lxr* | Forward | AGAGCTTCGTCCACAAAAGC | liver X receptor |
|  | Reverse | AGCACGTTGTAATAATGGAAGCC |  |
| *Npc1l1* | Forward | ATGTTACTCGCGTGGTTCAG | niemann-pick C1 like 1 |
|  | Reverse | AGGCCTTCTCTGCAAAACTG |  |
| *Shp1* | Forward | CGGACTTCCTTGCTTTGGATAG | small heterodimer partner 1 |
|  | Reverse | ATTTTGGCCTGGAGGTTTGG |  |
| *Srb1* | Forward | TTGGCCTGTTTGTTGGGATG | scavenger receptor class B type 1 |
|  | Reverse | TGCTGAGTCCGTTCCATTTG |  |
| *Srebf2* | Forward | TGCACCAGAGAGCATTTTGC | sterol regulatory element binding factor 2 |
|  | Reverse | AGGAACAAAGATGCCACAGC |  |
| *Gapdh* | Forward | ATGACTCTACCCACGGCAAG | glyceraldehyde 3-phosphate dehydrogenase |
|  | Reverse | TACTCAGCACCAGCATCACC |  |
